# Combined cortical thickness and blink reflex recovery cycle to differentiate essential tremor with and without resting tremor

**DOI:** 10.3389/fneur.2024.1372262

**Published:** 2024-02-23

**Authors:** Camilla Calomino, Andrea Quattrone, Maria Giovanna Bianco, Rita Nisticò, Jolanda Buonocore, Marianna Crasà, Maria Grazia Vaccaro, Alessia Sarica, Aldo Quattrone

**Affiliations:** ^1^Neuroscience Research Center, Department of Medical and Surgical Sciences, Magna Graecia University, Catanzaro, Italy; ^2^Department of Medical and Surgical Sciences, Institute of Neurology, Magna Graecia University, Catanzaro, Italy

**Keywords:** essential tremor, essential tremor plus, rest tremor, machine learning, blink reflex, cortical thickness

## Abstract

**Objective:**

To investigate the performance of structural MRI cortical and subcortical morphometric data combined with blink-reflex recovery cycle (BRrc) values using machine learning (ML) models in distinguishing between essential tremor (ET) with resting tremor (rET) and classic ET.

**Methods:**

We enrolled 47 ET, 43 rET patients and 45 healthy controls (HC). All participants underwent brain 3 T-MRI and BRrc examination at different interstimulus intervals (ISIs, 100–300 msec). MRI data (cortical thickness, volumes, surface area, roughness, mean curvature and subcortical volumes) were extracted using Freesurfer on T1-weighted images. We employed two decision tree-based ML classification algorithms (eXtreme Gradient Boosting [XGBoost] and Random Forest) combining MRI data and BRrc values to differentiate between rET and ET patients.

**Results:**

ML models based exclusively on MRI features reached acceptable performance (AUC: 0.85–0.86) in differentiating rET from ET patients and from HC. Similar performances were obtained by ML models based on BRrc data (AUC: 0.81–0.82 in rET vs. ET and AUC: 0.88–0.89 in rET vs. HC). ML models combining imaging data (cortical thickness, surface, roughness, and mean curvature) together with BRrc values showed the highest classification performance in distinguishing between rET and ET patients, reaching AUC of 0.94 ± 0.05. The improvement in classification performances when BRrc data were added to imaging features was confirmed by both ML algorithms.

**Conclusion:**

This study highlights the usefulness of adding a simple electrophysiological assessment such as BRrc to MRI cortical morphometric features for accurately distinguishing rET from ET patients, paving the way for a better classification of these ET syndromes.

## Introduction

Essential tremor (ET) is one of the most common neurological disorders, characterized by bilateral action tremor in the upper limbs, with or without tremor in other body segments, such as the head, jaw, torso or lower limbs (1, 2). ET patients typically present with kinetic and postural hand tremor, whereas tremor at rest is more suggestive of Parkinson’s disease (PD) (1, 3). Resting tremor, however, in the absence of overt bradykinesia or rigidity, has been reported also in a large percentage of ET patients (4), and these ET patients with resting tremor (rET) are now included into a diagnostic category termed “ET plus,” referring to ET patients with additional subtle motor or non-motor features (1, 5). This distinction allows keeping the ET category as “pure” as possible and may lead to more homogenous patient cohorts in research studies and trials, hopefully improving the research on these common tremor syndromes (1). Recent cohort studies provided evidence that ET plus may have a prevalence even higher than classic ET, and resting tremor is one of the most common symptoms in ET plus patients (4, 6–11). The clinical differential diagnosis between ET and rET is guided by the presence/absence of tremor at rest, but it may be challenging in several cases. In ET patients, resting tremor is usually not the main complaint, can fluctuate over time and is at times of low amplitude or even barely visible in the absence of electrophysiological analysis. A recent cohort study (7) including about 200 ET patients, showed that the diagnosis switched multiple times over a 5-year follow-up from ET to ET plus and vice versa in a significant percentage of patients, with resting tremor being the most unstable clinical feature. Around 40% of patients who received a clinical diagnosis of rET were classified as “pure” ET at follow-up visits and in some of them the diagnosis switched back again to rET later on since the resting tremor was not always present during consultations (7), making an accurate clinical differential diagnosis between ET and rET at times challenging.

A few studies explored the potential of paraclinical diagnostic procedures to support the differential diagnosis between ET patients with and without resting tremor (12, 13). From the neurophysiological perspective, the Blink reflex recovery cycle (BRrc), which is a simple test exploring the brainstem interneuron excitability (14–16), may be useful to this purpose. The blink reflex can be elicited by stimulating the supraorbital branch of the trigeminal nerve and is composed by an early, homolateral response (Rl) followed by a late, bilateral response (R2) (14–16). In healthy subjects, when the R2 response is evocated twice by electrical stimuli of equal intensity, the second R2 signal (R2-BRrc) is strongly inhibited at short interstimulus intervals (ISIs) of 100, 150, and 200 ms, while it recovers at longer ISIs (500–700 ms) (14, 16–20). In movement disorders, facial reflexes with short latencies such as the R1 of the blink reflex are typically normal, since the afferent and efferent fibers of the reflex arc are not affected by these diseases (14, 16, 21). On the contrary, reflexes with longer latencies and polysynaptic pathways are often altered in patients with movement disorders (i.e., dystonia, chorea, parkinsonian syndromes), since basal ganglia network alterations may produce hyper- or hypo-excitability of the brainstem inter-neuronal pool (14, 16, 21, 22). In the context of ET syndromes, a previous pilot study showed that R2-BRrc was enhanced at 150–200 ms in patients with rET but normal in ET patients and controls (19), thus suggesting a role of the R2-BRrc at short ISIs in distinguishing between these ET phenotypes.

In addition to neurophysiology, brain MRI is also an examination available in most centers and often included in the diagnostic work-up of patients with tremor syndromes (23). A plethora of imaging studies focused on MRI in ET patients (24–27) but there are only few reports investigating MR imaging differences between rET and ET patients (28–34). In a recent study, we developed a machine learning (ML) model based on structural cortical MRI data which differentiated these two patient groups with a mean AUC of 0.86 ± 0.11 (31). In the current study, we investigated whether adding R2-BRrc data to MRI morphometric features could further improve the performances in distinguishing between these two ET syndromes.

We employed two modern and well recognized ML decision tree-based classification algorithms [eXtreme Gradient Boosting (XGBoost) (35) and Random Forest (RF) (36)], with two specific aims: (i) to compare the classification performance of structural MR imaging data with those of the R2-BRrc in distinguishing between rET and ET patients, and (ii) to investigate whether a combination of R2-BRrc and imaging data may improve the classification of rET and ET patients.

## Materials and methods

### Subjects

Ninety ET patients (43 with and 47 without resting tremor) and 45 healthy controls (HC) were included in this study. Patients and controls were recruited at the Institute of Neurology and the Neuroscience Research Center of the Magna Graecia University, Catanzaro, Italy between 2017 and 2023. All patients underwent a detailed neurological examination performed by movement disorder specialists, and the clinical diagnosis of ET or rET was performed according to the recent diagnostic criteria of the Movement Disorder Society task force (1). Moreover, to provide the highest degree of diagnostic certainty *in vivo*, all patients included in the study underwent surface electromyographic tremor analysis as previously described (37, 38) to confirm or exclude the presence of resting tremor, and single photon emission computed tomography with 123I-ioflupane (DaTscan), performed as previously described (38), to rule out parkinsonian syndromes. A battery of cognitive tests was administered by an experienced neuropsychologist, including the Mini Mental State Examinations (MMSE) for general cognitive impairment (39), the Rey Auditory Verbal Learning Test immediate (RAVLT_I) and delayed recalls (RAVLT_D) for verbal learning and memory, the Controlled Oral Word Association Test (COWAT) for lexical stock, the Digit Span Forwards (Digit Span F) and Backwards (Digit Span B) for short-term verbal memory. The inclusion criterium for all study participants was the availability of BRrc recording and MR images for processing/analysis. Exclusion criteria were: possible dysmetabolic causes of tremor, DaTscan abnormal or not available, diffuse brain vascular lesions or basal ganglia/brainstem lesions on MRI, and ongoing or previous therapy with medications known to exacerbate or cause tremor, including amiodarone, amphetamines, beta-adrenergic agonists, antipsychotics, prednisone, lithium, and valproate. None of control subjects had any history of neurological, psychiatric, or major medical illnesses.

Some rET patients (*n* = 30), ET patients (*n* = 33) and the HC subjects were included in a previous study (31) investigating the performance of structural MRI data alone in differentiating between rET and ET patients. The final rET and ET groups, however, partially diverged from the previous study (31) due to the inclusion of newly enrolled patients and the exclusion of patients with R2-BRrc not available for prospective analysis. All study procedures and ethical aspects were approved by the institutional review board (Magna Graecia University review board, Catanzaro, Italy). Written informed consent for the research was obtained from all the individuals participating in the study.

### Blink reflex recovery cycle examination

All study participants underwent a blink reflex recovery cycle (BRrc) recording performed in accordance with previously described procedures (17, 19) on the same day of brain MRI and clinical examination. In brief, subjects were studied at rest, with eyes gently closed; the BRrc recording was performed delivering stimuli of 0.2 ms duration to the supraorbital nerve, and the muscular responses at the level of ipsilateral and contralateral orbicularis oculi muscles were recorded using surface electrodes below the eyelid and on the temple. Several stimuli intensities (between 5 and 30 mA) were explored, choosing the intensity which was three times the threshold of the blink reflex response. The blink reflex response to paired stimulation was assessed at interstimulus intervals (ISIs) of 100, 150, 200, and 300 ms. The area ratio of the R2 component of BRrc (R2 area of the conditioned response divided by the R2 area of the unconditioned response) was calculated for each ISI. The stimulation was performed bilaterally, but no significant differences were observed between the R2-BRrc values obtained from the right and left sides at each explored ISI; thus, for the purposes of our analysis, the measurements obtained from the right side were considered in all subjects. All medications with the potential to interfere with R2-BRrc were discontinued 2 days before the examination. Electrophysiological assessments were conducted and interpreted by an experienced technician who was unaware of the patients’ diagnoses.

### MRI acquisition and image processing

All study participants underwent a brain MR with a 3 T-MR750 GE MRI scanner (8-channel head coil), with a recently described protocol (40). Automated neuroanatomical segmentation was conducted using FreeSurfer 7.0 software (developed by Massachusetts General Hospital, Harvard Medical School; available at: http://surfer.nmr.mgh.harvard.edu) for all study participants. The standard “recon-all” pipeline (41) was employed for cortical reconstruction, and the Desikan–Killiany atlas was employed to delineate 34 cortical regions of interest (ROIs) for each hemisphere, with the subsequent automated computation of various morphometric metrics. These metrics included cortical thickness (CT), surface area (SA), cortical volume (CV), mean curvature (MC), and roughness (RG), this latter feature representing the standard deviation of cortical thickness (41, 42). Volumetric data for subcortical structures (subcortical volumes, SV) including the cerebellum, thalamus, caudate, putamen, globus pallidus, hippocampus, amygdala, and nucleus accumbens volumes were also extracted. Thus, a total of 358 structural MRI features were obtained for each subject.

### Statistical analysis

We assessed the normality of data distribution using the Shapiro–Wilk test. Differences in sex distribution were evaluated using Fisher’s exact test. To compare age at examination, age at disease onset, disease duration, education level and MMSE among subjects, we employed Kruskal-Wallis test followed by Dwass-Steel-Critchlow-Fligner pairwise comparison. Cognitive scores and imaging data were compared among groups with an analysis of covariance (ANCOVA) with covariates including age, sex, and education level (and intracranial volume for subcortical volumes). Linear correlations between structural imaging metrics and clinical/electrophysiological data were evaluated using Spearman’s correlation test. All *p* values were adjusted for multiple comparisons using Bonferroni’s correction, and a significance level of *p* < 0.05 was adopted. Statistical analyses were conducted using R statistical software (R for Unix/Linux, version 4.1.2, the R Foundation for Statistical Computing, 2014).

### Machine learning models

In this study, we employed Machine Learning (ML) models with two alternative algorithms based on decision trees [Random Forest (RF) (36) and XGBoost (35)] using structural MR imaging data and or R2-BRrc neurophysiological data, in distinguishing ET from HC, rET from HC, and rET from ET patients. MRI data included several cortical and subcortical features (CT, SA, CV, MC, RG, SV), while R2-BRrc data included the R2 area ratio at different ISIs. First, we investigated the performances of ML model using either MRI or R2-BRrc data separately; second, we tested a model combining imaging and neurophysiologic data, aiming to increase the classification performance. For each model, the hyperparameters were tuned through five-fold cross-validation (cv) with randomized search (10 iterations) to maximize the accuracy, splitting the dataset into training (80%) and validation folds (20%). In detail, we divided the dataset into K subsets (folds) and iteratively trained the model K times. For each iteration, the model was trained on (K-1) folds and validated on the Kth fold, which was not used to train the model. The hyperparameters tuned for XGB were: learning rate, maximum depth, minimum child weight, gamma and colsample bytree (the fraction of features [randomly selected] that will be used to train each tree) (31, 43). The hyperparameters tuned for RF were: number of trees in the forest, max number of features considered for splitting a node, max number of levels in each decision tree, min number of data points placed in a node before the node is split, min number of data points allowed in a leaf node and bootstrap a method for sampling data points (31, 44). The feature importance was evaluated through the “permutation accuracy importance” technique (45), assessing the Mean Decrease in Accuracy after permuting each feature, using 50 repetitions to ensure the reliability of the feature ranking. Subsequently, a feature selection procedure was applied by iteratively training the models on the features ordered according to their importance. Finally, the performance of the tuned XGB and RF models trained on the most important features were evaluated in five-fold cross-validation analysis (80–20 split), repeated 5 times, and the mean and standard deviation of area under the curve (AUC), accuracy, sensitivity and specificity in all 25 validation folds were calculated. A model was considered able to distinguish between groups when the mean AUC was >0.85.

To evaluate whether of our top-performing models were prone to possible overfitting, which may occur when ML approaches are used in relatively small sample sets, we performed a permutation analysis as previously described (46). All patients from the original dataset were randomly assigned to either the “rET,” or “ET” groups, by switching randomly the clinical labels, and our best ML model was trained on this new artificial dataset (46, 47). This permutation strategy was repeated 100 times, and the average AUC of our best model was calculated. By changing the clinical labels, we could remove the biological differences between groups, creating a “random” dataset with feature values in the range of the real values. On these bases, a classification performance drops to around 50% in permutation analysis is expected, while a persistently high AUC may raise concerns of model overfitting the data beyond biological differences (46, 47). The analyses were conducted with Python 3.9 and the packages scikit-learn v1.0.2.

## Results

### Demographic, clinical, and electrophysiological data

The demographic and clinical data of patients and controls are reported in Table 1. There were no differences in demographic variables (age at examination, sex, education level) among groups. Both rET patients had slightly lower MMSE scores than HC, without any difference in other domain-specific tests, and without differences between rET and ET groups. rET patients had significantly higher R2-BRrc area ratio values at ISIs 150, 200, and 300 ms than ET and HC, while no differences were found between ET and HC. At ISI of 100 ms, no differences in R2-BRrc area ratio values were found among the three groups, with completely inhibited response in HC and very low values in both patient groups.

**Table 1:** Demographic, clinical and electrophysiological data of patients with classical essential tremor, patients with essential tremor with resting tremor, and healthy control subjects.

Data	rET (*N* = 43)	ET (*N* = 47)	HC (*N* = 45)	*p*-value
Sex, (M/F)	21/22	28/19	18/27	0.403^a^
Age at examination, ys^b^	63.7 ± 11.2	65.1 ± 9.7	68.5 ± 6.9	0.170^c^
Disease onset, ys^b^	47.1 ± 17.1	51.8 ± 17.6	–	0.169^c^
Disease duration, ys^b^	17.4 ± 14.1	13.3 ± 15.3	–	0.260^c^
Education level, ys^b^	8.68 ± 5.02	11.2 ± 4.90	10.9 ± 4.29	0.128^c^
MMSE^b^	26.0 ± 2.61	26.70 ± 2.54	27.6 ± 2.14	**0.040** ^c^^,*^
COWAT^b^	22.7 ± 6.75	25.6 ± 6.22	26.1 ± 10.2	0.786^d^
RAVLT-I^b^	34.84 ± 9.66	36.97 ± 9.33	34.2 ± 8.34	0.604^d^
RAVLT-D^b^	6.94 ± 2.80	6.81 ± 2.64	6.18 ± 2.43	0.463^d^
Digit Span Forwards^b^	4.91 ± 0.81	5.77 ± 3.83	5.07 ± 0.87	0.808^d^
Digit Span Backwards^b^	3.00 ± 0.68	3.44 ± 0.88	3.3 ± 0.82	0.882^d^
R2BRrc area ratio – ISI 100^b^	8.21 ± 2.58	2.53 ± 1.56	0	0.063^d^
R2BRrc area ratio – ISI 150^b^	24.56 ± 3.28	3.6 ± 2.0	0	**7.48e-7**^d^^,*,^°
R2BRrc area ratio – ISI 200^b^	36.51 ± 4.10	4.30 ± 2.26	0	**2.73e-9**^d^^,*,^°
R2BRrc area ratio – ISI 300^b^	49.47 ± 4.64	19.57 ± 3.12	1.51 ± 5.94	**9.59e-7**^d^^,*,^°

ET, essential tremor; rET, essential tremor with resting Tremor; HC, healthy controls; MMSE, Mini Mental State Examination; COWAT, Controlled Oral Word Association Test; RAVLT-I, Rey Auditory Verbal Learning Test immediate recall; RAVLT-D, Rey Auditory Verbal Learning Test delayed recall.

The full cognitive battery was available in 59 participants (19 ET, 20 rET, 19 HC).

All rET patients had rest tremor; regarding other soft signs, 11 patients had subtle parkinsonian signs, 10 patients had mild memory deficits, 9 patients had impaired tandem gait and 3 patients had questionable dystonic posturing.

a Fishers exact test.

b Data are expressed as mean ± standard deviation.

c Kruskal-Wallis test followed by Dwass-Steel-Critchlow-Fligner pairwise comparison.

d ANCOVA followed by *post-hoc* test (covariates: age, sex, education level).

Significant *p*-values are highlighted in bold.

^*^rET vs. HC *p*-value < 0.05.

°rET vs. ET *p*-value < 0.05.

^#^ET vs. HC *p*-value < 0.05.

### MRI cortical and subcortical morphometric features

As showed in Supplementary Table S1, ET patients with resting tremor (rET) had increased roughness in the right lateral orbito-frontal cortex in comparison with HC, while classic ET patients showed increased roughness in the right superior temporal cortex and reduced mean curvature in the right rostral anterior cingulate cortex compared with HC. By directly comparing the two ET groups (Supplementary Table S1), rET patients showed increased roughness and mean curvature in temporal regions and reduced surface area in the right precentral cortex (frontal lobe). The temporal lobe regions showing increased roughness or mean curvature in rET patients compared to classic ET, showed significant negative correlations with COWAT test in the rET group (Supplementary Table S2). On the contrary, no correlations were found between these imaging variables and age, disease duration, education level and R2BRrc data. In contrast to cortical regions, no differences were observed in the volumes of subcortical structures across the three groups.

### Classification performance of ML models in distinguishing patients from controls

**Figure 1:**
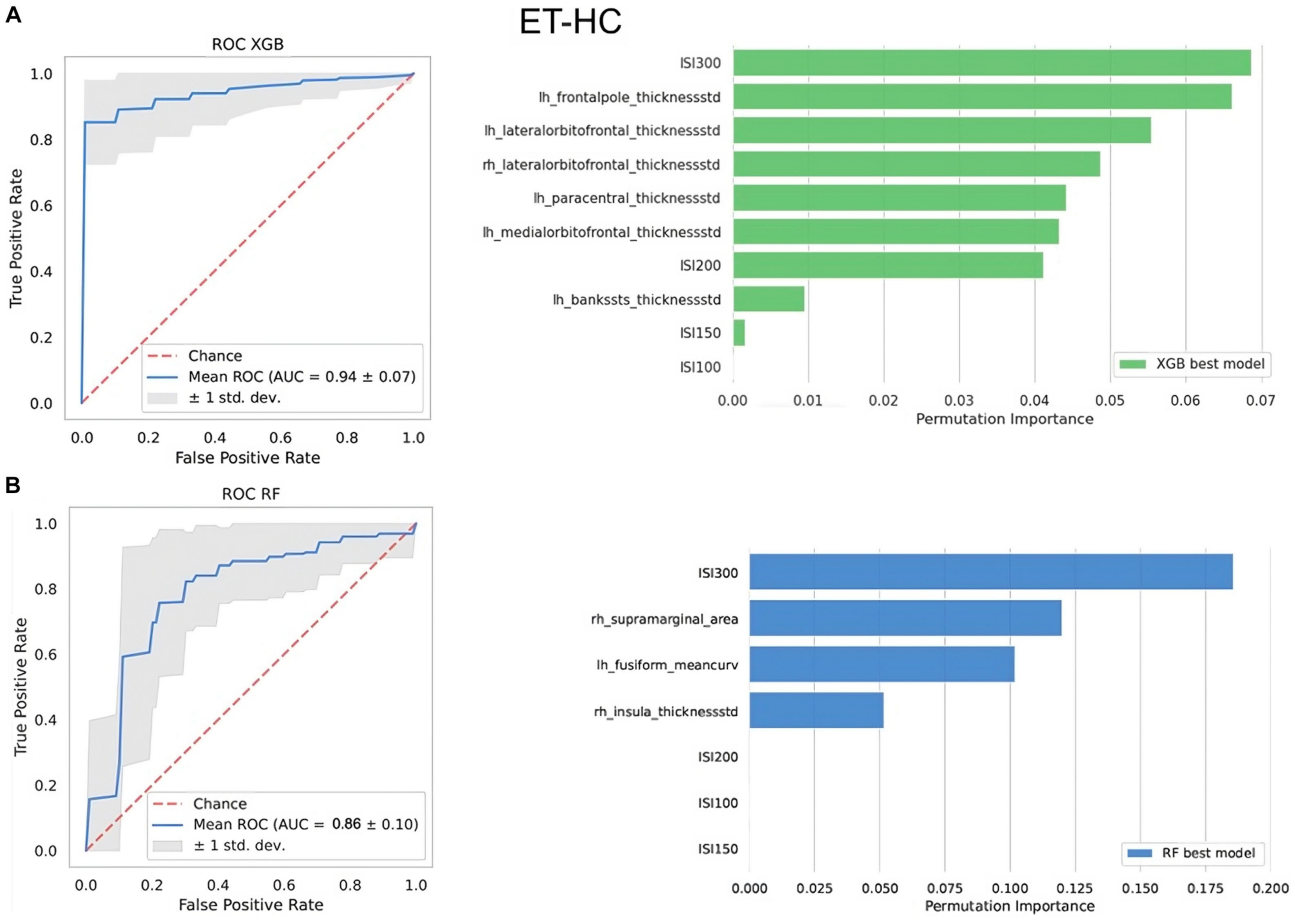
The top-performing machine learning models in differentiating between ET patients and healthy controls. At the top, the best XGBoost model **(A)**; at the bottom, the best Random Forest model **(B)**. On the left side of the figure, it is shown the ROC curve. On the right side, it is shown the relative importance of the features selected by the model in distinguishing between the two groups assessed via permutation method. Features are shown in descending order from the most to the less important feature. Rh, right; Lh, left; AUC, area under the curve; thicknessstd, standard deviation of thickness, which is the roughness; ISI, interstimulus interval.

In the ET vs. HC classification task, neither ML models using MRI features without R2-BRrc, nor those using combinations of R2-BRrc data without MRI, reached acceptable AUC values (Table 2). With both algorithms, the performance increased when imaging and neurophysiologic data were used together as input for ML models (Table 2), and the best model in distinguishing ET from HC used XGBoost algorithm on cortical roughness and R2-BRrc area ratio at various ISIs, reaching AUC of 0.94 ± 0.07 in cross-validation analysis (Table 2; Figure 1).

**Table 2:** Classification performance of machine learning models with XGBoost and random forest and on R2-BRrc and MR imaging data, in distinguishing between patients with essential tremor with and without rest tremor and healthy controls.

Models	MRI	R2-BRrc	MRI + R2-BRrc
**ET vs. HC**			
XGBoost	AUC: 0.821 (0.106)	AUC: 0.797 (0.049)	AUC: 0.940 (0.070)
	ACC: 0.770 (0.088)	ACC: 0.782 (0.049)	ACC: 0.882 (0.034)
	SENS: 0.810 (0.140)	SENS: 0.640 (0.071)	SENS: 0.800 (0.080)
	SPEC: 0.729 (0.130)	SPEC: 0.940 (0.089)	SPEC: 0.950 (0.023)
	(#9)	(#1)	(#10)
Random forest	AUC: 0.761 (0.089)	AUC: 0.780 (0.084)	AUC: 0.860 (0.010)
	ACC: 0.720 (0.091)	ACC: 0.783 (0.083)	ACC: 0.783 (0.083)
	SENS: 0.767 (0.114)	SENS: 0.640 (0.108)	SENS: 0.640 (0.108)
	SPEC: 0.671 (0.152)	SPEC: 0.933 (0.089)	SPEC: 0.933 (0.089)
	(#3)	(#1)	(#4)
**rET vs. HC**			
XGBoost	AUC: 0.855 (0.130)	AUC: 0.889 (0.052)	AUC: 0.916 (0.053)
	ACC: 0.833 (0.123)	ACC: 0.853 (0.050)	ACC: 0.861 (0.057)
	SENS: 0.822 (0.174)	SENS: 0.877 (0.131)	SENS: 0.777 (0.136)
	SPEC: 0.844 (0.122)	SPEC: 0.933 (0.070)	SPEC: 0.942 (0.071)
	(#6)	(#1)	(#6)
Random forest	AUC: 0.635 (0.127)	AUC: 0.881 (0.050)	AUC: 0.912 (0.061)
	ACC: 0.602 (0.096)	ACC: 0.848 (0.050)	ACC: 0.866 (0.054)
	SENS: 0.523 (0.179)	SENS: 0.749 (0.120)	SENS: 0.777 (0.131)
	SPEC: 0.676 (0.172)	SPEC: 0.832 (0.070)	SPEC: 0.951 (0.063)
	(#4)	(#1)	(#5)
**rET vs. ET**			
XGBoost	AUC: 0.858 (0.075)	AUC: 0.815 (0.079)	AUC: 0.941 (0.052)
	ACC: 0.780 (0.099)	ACC: 0.822 (0.075)	ACC: 0.882 (0.081)
	SENS: 0.751 (0.165)	SENS: 0.697 (0.123)	SENS: 0.860 (0.093)
	SPEC: 0.802 (0.113)	SPEC: 0.936 (0.086)	SPEC: 0.901 (0.098)
	(#12)	(#1)	(#12)
Random forest	AUC: 0.696 (0.136)	AUC: 0.821 (0.080)	AUC: 0.872 (0.073)
	ACC: 0.662 (0.121)	ACC: 0.822 (0.075)	ACC: 0.820 (0.084)
	SENS: 0.585 (0.198)	SENS: 0.697 (0.123)	SENS: 0.818 (0.108)
	SPEC: 0.731 (0.155)	SPEC: 0.936 (0.086)	SPEC: 0.823 (0.125)
	(#13)	(#1)	(#3)

ET, essential tremor; rET, essential tremor with resting tremor; HC, healthy controls; AUC, Area Under the Curve, ACC, accuracy; SENS, sensitivity; SPEC, specificity. The classification performances are shown as mean (standard deviation) in the repeated 5-fold cross-validation folds. The number of features used by each model determined using the feature selection is reported in round brackets (#). Data shown in the table refer to the best model for each feature group.

**Figure 2:**
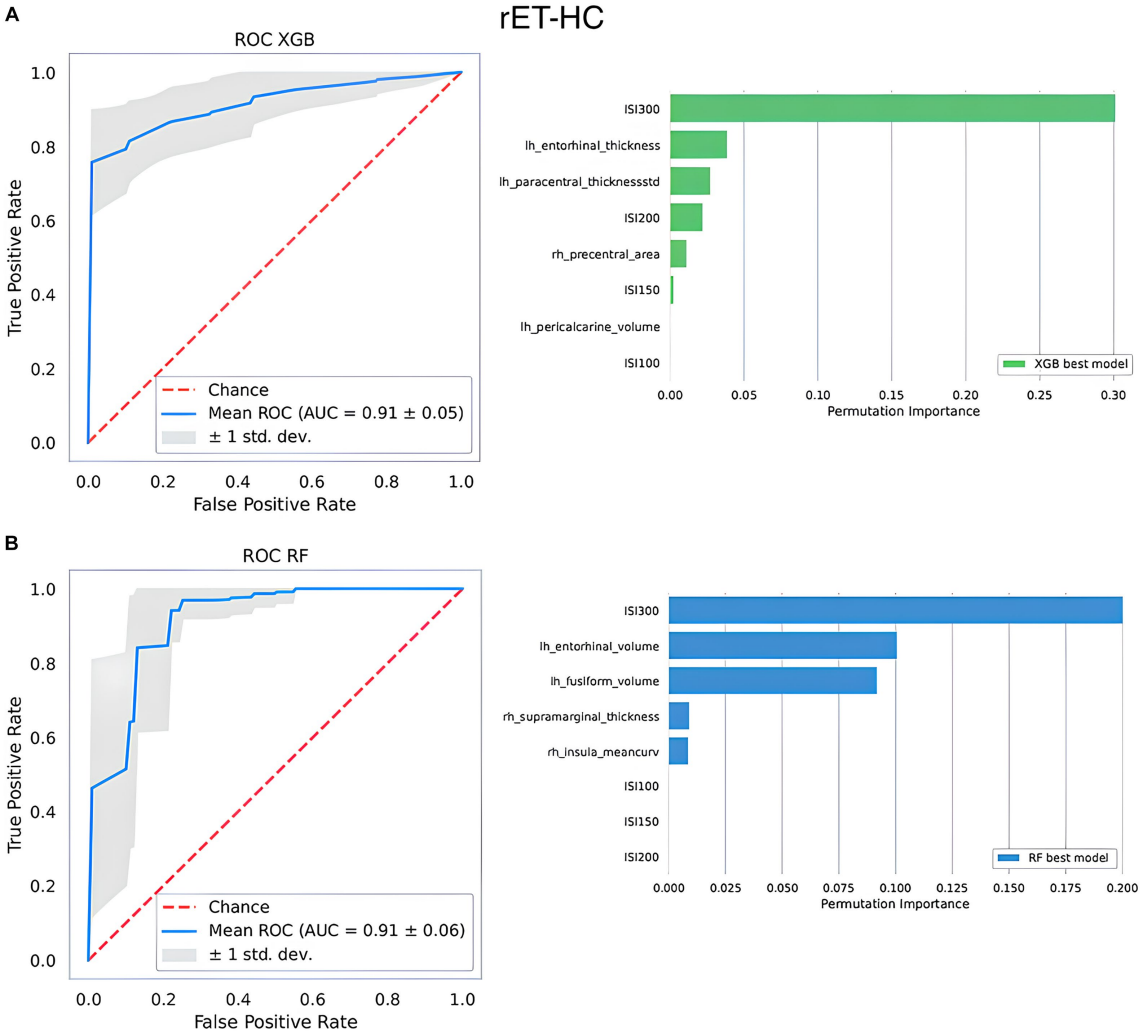
The top-performing machine learning models in differentiating between rET patients and healthy controls. At the top, the best XGBoost model **(A)**; at the bottom, the best Random Forest model **(B)**. On the left side of the figure, it is shown the ROC curve. On the right side, it is shown the relative importance of the features selected by the model in distinguishing between the two groups assessed via permutation methods. Features are shown in descending order from the most to the less important feature. Rh, right; Lh, left; AUC, area under the curve; thicknessstd, standard deviation of thickness, which is the roughness; ISI, interstimulus interval.

In the rET vs. HC classification task, ML models using R2-BRrc data only yielded acceptable performances (AUC around 0.88 both using XGBoost or RF) and outperformed ML models based on MRI only (barely acceptable performance with XGBoost [AUC: 0.85 ± 0.13], and very low performance with RF [AUC: 0.63 ± 0.13]), as shown in Table 2. An increase in AUC values, however, was observed with models combining MRI and R2-BRrc data (AUC: 0.91 ± 0.05, both with XGBoost and RF). The best models with feature importance analysis are shown in Figure 2.

### Classification performance of ML models in distinguishing between ET groups

**Figure 3:**
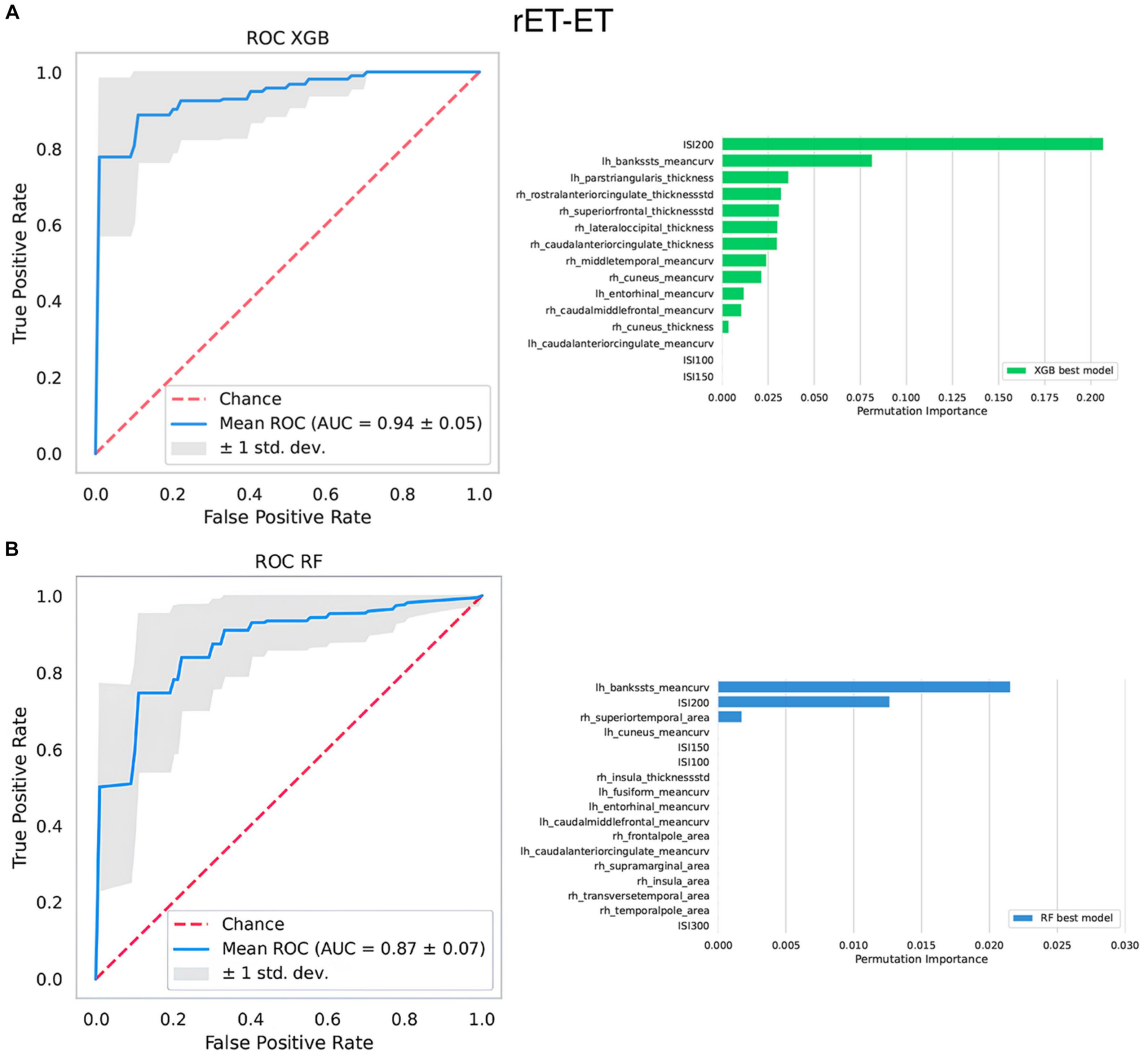
The top-performing machine learning models in differentiating between ET patients and rET patients. At the top, the best XGBoost model **(A)**; at the bottom, the best Random Forest model **(B)**. On the left side of the figure, it is shown the ROC curve. On the right side, it is shown the relative importance of the features selected by the model in distinguishing between the two groups assessed via permutation methods. Features are shown in descending order from the most to the less important feature. Rh, right; Lh, left; AUC, area under the curve; thicknessstd, standard deviation of thickness, which is the roughness; ISI, interstimulus interval.

Similar to the previous classification tasks, the two algorithms reached almost identical performances when using R2-BRrc data, while XGBoost outperformed RF with MRI data. Thus, the superiority of models using selectively either MRI or R2-BRrc data in distinguishing the two ET groups varied depending on the algorithm employed: MRI-based models were more powerful than R2-BRrc-based models when XGBoost was used and vice versa with RF (Tables 2, 3). The best model using MRI only reached AUC: 0.86 ± 0.07 (XGBoost), while the best models using R2-BRrc data obtained AUC values around 0.81–0.82 (Table 2). Both algorithms showed a significant improvement in the classification performance when neurophysiologic data were added to the MR imaging features as input for ML models (Tables 2, 3). The performance of XGBoost best model raised from AUC: 0.86 ± 0.07 (obtained using MRI only) to AUC: 0.94 ± 0.05 (obtained using MRI and R2-BRrc data) in distinguishing between rET and ET patients (Table 2). The same phenomenon was confirmed by RF models, whose AUC raised from 0.70 ± 0.14 (MRI only) to AUC: 0.87 ± 0.07 (MRI and R2-BRrc data). The feature importance analysis identified the R2-BRrc at ISI 200 and the mean curvature in temporal regions as the most informative features for classification between ET groups in both XGBoost and RF best models (Figure 3).

**Table 3:** Statistical differences among the performances of different models in distinguishing between patients with essential tremor with and without rest tremor.

	Data	MRI	R2-BRrc	Combined model
XGBoost	MRI	–	*p* = 0.021	*p* = 0.001
	R2-BRrc	–	–	*p* < 0.001
Random forest	MRI	–	*p* = 0.003	*p* < 0.001
	R2-BRrc	–	–	*p* = 0.01

The combined models included MRI models and R2-BRrc. The performances were compared across different models using Friedman test followed by the Durbin-Conover *post-hoc* for pairwise comparisons.

### Permutation analysis on the best ML models

The permutation analysis performed through random re-classification of patients repeated 100 times showed a marked drop in the classification performances of the best models for each classification task, demonstrating that our models did not significantly overfit the data beyond biological differences. In detail, the best model for classification between rET and ET showed a drop in the AUC from 0.94 to 0.61; similar results were found for the best models for rET vs. HC (AUC from 0.91 to 0.57) and ET vs. HC classification (AUC from 0.94 to 0.61).

## Discussion

In this study, we employed a ML approach based on different cortical and subcortical MRI morphometric measurements and R2-BRrc data to distinguish between patients with ET with and without rest tremor. This study demonstrated that both these techniques may be useful in the differential diagnosis between these two ET syndromes. Moreover, the addition of BRrc data to MRI features in ML models can significantly improve the classification performances, suggesting a complementary diagnostic value of these two diagnostic procedures and enabling a better classification of ET patients.

The recent consensus statement of tremors included ET patients with rest tremor (rET) into a category termed “ET plus” referring to ET patients with additional motor or non-motor features (rest tremor, impaired tandem gait, cognitive impairment, questionable dystonic posturing or subtle parkinsonian features) (1). At the present time, however, it is not yet known whether these two ET phenotypes (classical ET and ET plus) are either part of a continuum or two separated entities (6, 12, 48–50). Some authors suggested that rest tremor could represent a late clinical feature of ET, while others argued that rET may be a disorder distinct from ET, with higher age at disease onset or a transitional state between ET and PD (6, 12, 48–50). On these bases, it is key to explore similarities and differences between ET and ET plus patients to improve the knowledge of these tremor syndromes.

The current study is one of the few reports assessing MRI differences between different ET syndromes and revealed a slight cortical involvement characterized by increased roughness and mean curvature and reduced surface area in some fronto-temporal regions in rET patients compared to those with ET and HC, correlating with cognitive scores. These differences were identified since we employed advanced surface-based techniques capable of estimating multiple complementary morphometric characteristics of cortical structures, providing additional insights into brain structure and detecting even subtle cortical alterations (41, 42, 51–53). We indeed examined various cortical metrics, encompassing not only the well-established parameters like cortical thickness and volume (which revealed no differences between ET and rET), but also surface area, roughness and mean curvature, which may be more sensitive indicators of cortical atrophy (51–53). Roughness, a recently introduced metric, is computed as the standard deviation of cortical thickness, and an increase in this metric implies some degree of cortical atrophy (52). Mean curvature values furnish a quantitative gage of cortical folding, with increased values indicating sharper cortical folds, which could signify cortical atrophy or subcortical white matter atrophy (51). In addition to structural cortical differences, previous functional MRI studies have also identified differences between these two ET syndromes in cortical regions using different techniques. A resting-state MRI study (33) revealed reduced neural activity in secondary motor cortex regions (right superior and middle frontal gyrus, right precentral gyrus, and right Supplementary motor area) in rET patients compared with ET patients, and another study (29) reported decreased activation in parietal areas in rET compared to ET patients. Differently from cortical regions, we did not find any differences between rET and ET patients in the volumes of subcortical structures. These results are in line with previous studies showing no differences in the degree of cerebellar involvement between these ET syndromes (25, 28, 29, 32, 34); on the other hand, some diffusor tensor imaging (DTI) studies exploring white matter integrity suggested the implication of basal ganglia circuits in the physiopathology of rET (29, 30), while classic ET syndrome did not show the involvement of these structures, leading to the hypothesis that rest tremor associated with ET might be linked to basal ganglia dysfunction. It is possible to hypothesize that MR volumes may be not sensitive enough, (i.e., less than DTI) in detecting such differences.

In a recent previous study (31), we found that subtle cortical MRI differences between rET and ET could lead to acceptable classification performances (AUC of 0.86 ± 0.11) in distinguishing between these syndromes leveraging modern ML algorithms (XGboost). Here, we investigated whether the addition of R2-BRrc data to MRI morphometric features could further improve the performances in distinguishing between these two ET syndromes. The current study expands our previous work in several ways: (i) demonstrating an overt superiority of XGBoost over RF algorithms when dealing with morphometric MRI data such as thickness, volumes, roughness, surface area and mean curvature in all comparisons, (ii) allowing to accurately distinguish classic ET patients from HC, which was not possible by using MRI morphometric features alone, and most importantly (iii) improving the distinction between rET and ET patients (compared to ML models using MRI alone) without the drawback of additional costs or complexity; this was obtained through the addition of R2-BRrc test, which is an extremely simple, cheap and widely available neurophysiological procedure. The rationale of adding these neurophysiologic data was that the R2-BRrc area ratio at short ISIs has been reported to be higher in rET patients compared to ET in a previous pilot study (19). In this study, a ML approach based only on R2-BRrc area ratio at ISIs of 100, 150, 200, and 300 ms showed AUC of 0.81–0.82, confirming the potential usefulness of this diagnostic test in distinguishing rET from ET. When these neurophysiologic data were included together to MRI morphometric features in ML models, we found a significant increase in classification performances, with the best model achieving an excellent AUC of 0.94 ± 0.05 in distinguishing between the two ET syndromes. Of note, the R2-BRrc area ratio at ISI of 200 ms was one of the two most important features used for classification by the models (together with the mean curvature of temporal regions, which was also significant in ANCOVA), both with XGBoost and RF algorithms. This result proved that the R2-BRrc was indeed not a minor addition to the models, and highlighted the importance of coupling neurophysiologic and imaging data which may have a complementary diagnostic value. The accurate classification of rET and ET patients is of high relevance for several reasons. First, it can have strong implications for future research; this perfectly aligns with the purposes of the recent consensus statement on tremors by the Movement Disorders Society, which created the new diagnostic category of “ET plus” (including ET patients with rest tremor) to reduce heterogeneity in ET cohorts (1, 5, 12). The main advantage of this new approach is to recruit homogenous patient groups for clinical and translational research studies in ET, likely improving future understanding of this tremor syndrome (1, 5, 12). Moreover, a recent study (54) showed higher percentage of longitudinal clinical tremor worsening and lower responsiveness to anti-tremor medications in ET plus compared to “pure” ET patients, suggesting that an accurate classification of ET syndromes may have prognostic implications. Further studies, however, are needed to confirm these data.

Beyond patient classification, our study also provides insights on the highly debated relationship between these two syndromes, suggesting that rET is likely to be a distinct syndrome (or a different subtype of the same syndrome) rather than just a late stage of ET, with possible pathophysiological differences. Indeed, while the slightly higher cortical involvement in rET might suit with the “late stage” hypothesis, the enhanced brainstem excitability (reflected by higher R2-BRrc) observed in rET does not. In other conditions (i.e., PD or dystonia), the R2-BRrc is clearly enhanced already in the early stages of the disease (14, 20, 55); therefore, it may be a bit odd to hypothesize a completely opposite behavior in ET, with a R2-BRrc remaining normal for many years and becoming abnormal in the advanced stages of the disease. The BRrc alteration, however, is not pathognomonic of a single neurological entity, thus precluding a definitive association of rET syndrome with either Parkinson’s disease or dystonic syndromes. Future imaging or genetic studies exploring similarities and differences between rET patients and these other neurological disorders may shed more light on this point.

In the current study, the advantage of combining MRI and R2-BRrc data was confirmed by two distinct ML algorithms. By looking at the classification performances, XGBoost was more powerful than RF when using multiple MRI morphometric features. Both XGBoost and RF are tree-based ML algorithms sharing common rules for tree growth, but they diverge in their approach to constructing an ensemble of trees. RF employs a technique known as bagging to simultaneously construct multiple trees, followed by making predictions through a majority voting mechanism (36). On the contrary, XGB constructs a sequential ensemble of trees with the goal of enhancing the performance of each subsequent tree by rectifying the errors of the preceding one (35, 56). It is possible to hypothesize that the ability of XGBoost learn from its wrong predictions and correct its mistakes may be responsible for his generally higher classification performances (56), but this hypothesis is only speculative in nature, since these powerful ML algorithms function as “black boxes,” making decisions without providing supporting evidence.

Despite some discrepancy in the absolute performances, both algorithms, however, provided comparable results in terms of performance increase when the two sources of data were combined together, strengthening the reliability of our main findings and take-home messages.

The current study has several strengths. First, rET and ET clinical diagnoses were performed by a movement disorder specialist according to international diagnostic criteria (1); in addition, the clinical diagnosis was supported by electrophysiological tremor recording confirming/excluding the presence of rest tremor and by a normal DaTscan, thus ruling out Parkinson’s disease. Second, all MRI data were acquired through fully automated and validated procedures. Third, we employed two distinct ML algorithm and compared the results between them to enhance the robustness and reliability of our findings.

The main limitation to this study is the relatively small sample size and the lack an independent validation cohort; thus, future studies in large international patient cohorts are needed to demonstrate the generalizability of our results. Since the limited sample size coupled to a relatively large number of predictors may at times lead to data overfitting by ML models, in this study, we performed a permutation analysis to investigate/exclude the presence of data overfitting (46, 47). The classification accuracy dropped from above 0.90 to around 0.60 in permutation analysis, suggesting that the model was not able to correctly classify patients when the biological differences were artificially removed, thus demonstrating no significant overfitting by our ML models. A second limitation is the lack of post-mortem pathological examination. We used clinical and paraclinical investigation to reach the highest degree of diagnostic certainty *in vivo*, as described above; however, misdiagnosis may have occurred in a few cases; moreover, it is not possible to exclude that some ET patients may develop rest tremor and thus evolve to ET plus in the future. Third, this is a cross-sectional study; future longitudinal studies are warranted to investigate the possible usefulness of these models in predicting diagnosis refinement at follow-up (i.e., patients with ET diagnosis who may develop rest tremor over time). A final limitation to the immediate large use of our approach is the complexity of ML approaches; however, there has been a growing interest in artificial intelligence within the medical field, and classification models, often combining different types of biomarkers, are increasingly recognized as valuable tools for facilitating the differential diagnosis process and providing guidance in clinical decision-making (57–60); thus, this technology will likely find its place in clinical practice quite soon. In this context, the availability of T1-weighted MRI and simple low-cost neurophysiologic examinations such as R2-BRrc may significantly contribute to a widespread use of this biomarker. Indeed, though not mandatory for clinical diagnosis in patients with isolated tremor syndromes, structural MRI examination is often performed in patients with tremor to investigate/exclude the presence of lesions or atrophy in the basal ganglia, cerebellum and brainstem (23). The BRrc, due to its simplicity and low associated economic burden, could be considered a screening test in patients presenting with tremor providing hints in the differential diagnosis between “pure ET” and other tremor syndromes, to include in the protocols of neurophysiology tremor studies.

In conclusion, this study demonstrates that ML models combining structural MRI measurements of cortical regions and R2-BRrc values showed excellent performance in distinguishing rET from ET patients. This novel approach holds promise in facilitating the precise classification of ET patients, which is crucial for a better understanding of these still largely unexplored tremor syndromes.

## Data availability statement

The raw data supporting the conclusions of this article will be made available by the authors, without undue reservation.

## Ethics statement

The studies involving humans were approved by Magna Graecia University review board, Catanzaro, Italy. The studies were conducted in accordance with the local legislation and institutional requirements. The participants provided their written informed consent to participate in this study.

## Author contributions

CC: Conceptualization, Data curation, Formal analysis, Methodology, Writing – original draft. AnQ: Conceptualization, Supervision, Writing – original draft, Writing – review & editing. MB: Data curation, Formal analysis, Methodology, Supervision, Writing – review & editing. RN: Data curation, Writing – review & editing. JB: Data curation, Writing – review & editing. MC: Data curation, Writing – review & editing. MV: Data curation, Writing – review & editing. AS: Data curation, Methodology, Supervision, Writing – review & editing. AlQ: Conceptualization, Supervision, Writing – review & editing.
