# Supplementary material for: Combined cortical thickness and blink reflex recovery cycle to differentiate essential tremor with and without resting tremor
Source: Front Neurol. 2024 Feb 23;15:1372262. doi: 10.3389/fneur.2024.1372262 (PMC10995929; doi:10.3389/fneur.2024.1372262)
Supplement: Supplementary file 1 [file Table_1.DOCX]

**Supplementary Table 1.** MRI data of patients with essential tremor, patients with essential tremor with resting tremor and healthy control subjects.

|  |  |  |  |  | **ANCOVA post-hoc** | |
| --- | --- | --- | --- | --- | --- | --- |
| **Regions** | **Lobe** | **rET**  **(n=43)** | **ET**  **(n=47)** | **HC**  **(n=45)** | **Adjusted p-value** | **Pairwise comparisons** |
| ***ROUGHNESS*** |  |  |  |  |  |  |
| Lh parahippocampal | Temporal | 0.750 (0.020) | 0.700 (0.093) | 0.750 (0.011) | 0.04 | **rET > ET** |
| Rh lateral orbito-frontal | Frontal | 0.748 (0.012) | 0.715 (0.011) | 0.705 (0.012) | 0.04 | **rET > HC** |
| Rh superior temporal | Temporal | 0.603 (0.008) | 0.609 (0.008) | 0.583 (0.008) | 0.04 | **ET > HC** |
| ***MEAN CURVATURE*** |  |  |  |  |  |  |
| Lh entorhinal | Temporal | 0.121 (0.002) | 0.111 (0.002) | 0.116 (0.002) | 0.01 | **rET > ET** |
| Rh rostral anterior cingulate | Frontal | 0.132 (0.002) | 0.129 (0.002) | 0.136 (0.002) | 0.03 | **ET < HC** |
| ***SURFACE AREA*** |  |  |  |  |  |  |
| Rh precentral | Frontal | 4356 (69.1) | 4628 (64.3) | 4441 (65.4) | 0.01 | **rET < ET** |

Abbreviations: ET = essential tremor; rET = essential tremor with resting tremor; lh = left hemisphere; rh = right hemisphere.

The table shows structural MRI data obtained with FreeSurfer v7. Data are expressed as the mean ± the standard error.

ANCOVA was performed with age, sex and education level as covariates. Only significant results corrected for Bonferroni are showed.
