# Supplementary material for: Combined cortical thickness and blink reflex recovery cycle to differentiate essential tremor with and without resting tremor
Source: Front Neurol. 2024 Feb 23;15:1372262. doi: 10.3389/fneur.2024.1372262 (PMC10995929; doi:10.3389/fneur.2024.1372262)
Supplement: Supplementary file 2 [file Table_2.DOCX]

**Supplementary Table 2.** Significant correlations between MR imaging morphometric features and neuropsychiatric tests in patients with essential tremor with resting tremor.

| **Data** | Lh parahippocampal | Lh entorhinal | Rh precentral |
| --- | --- | --- | --- |
| MMSE | NS | NS | NS |
| COWAT | -0.48 (0.042) | -0.49 (0.040) | NS |
| RAVLT-I | NS | NS | NS |
| RAVLT-D | NS | NS | NS |
| Digit_span_Forwards | NS | NS | NS |
| DIGIT SPAN B | NS | NS | NS |

Abbreviations: Lh = left; Rh = right; MMSE = Mini Mental State Examination; COWAT = Controlled Oral Word Association Test; RAVLT-I = Rey Auditory Verbal Learning Test immediate recall; RAVLT-D = Rey Auditory Verbal Learning Test delayed recall. Results are expressed as Spearman’s rho correlation coefficient (p value). Possible correlations were investigated between cognitive scores and brain metrics significantly different between rET patients and ET patients.
